# Study protocol for reducing disparity in receipt of mother’s own milk in very low birth weight infants (ReDiMOM): a randomized trial to improve adherence to sustained maternal breast pump use

**DOI:** 10.1186/s12887-021-03088-y

**Published:** 2022-01-07

**Authors:** Tricia J. Johnson, Paula P. Meier, Michael E. Schoeny, Amelia Bucek, Judy E. Janes, Jesse J. Kwiek, John A. F. Zupancic, Sarah A. Keim, Aloka L. Patel

**Affiliations:** 1 Department of Health Systems Management, Rush University, 1700 West Van Buren Street, TOB Suite 126B, Chicago, USA; 2 Department of Pediatrics, Rush University Medical Center, Chicago, USA; 3 College of Nursing, Rush University, Chicago, USA; 4 Department of Community, Systems and Mental Health Nursing, Rush University, Chicago, USA; 5 Department of Microbiology, The Center for Retrovirus Research and the Infectious Disease Institute, The Ohio State University, Columbus, USA; 6 Department of Neonatology, Beth Israel Deaconess Medical Center, Boston, USA; 7 Harvard Medical School, Boston, USA; 8 Center for Biobehavioral Health, The Abigail Wexner Research Institute, Nationwide Children’s Hospital, Columbus, USA; 9 Department of Pediatrics, The Ohio State University College of Medicine, Columbus, USA; 10 Division of Epidemiology, The Ohio State University College of Public Health, Columbus, USA

**Keywords:** Very low birth weight, Very preterm, Neonatal intensive care unit, Mother’s own milk, Maternal breast milk, Economic evaluation, Cost-effectiveness analysis

## Abstract

**Background:**

Black very low birth weight (VLBW; < 1500 g birth weight) and very preterm (VP, < 32 weeks gestational age, inclusive of extremely preterm, < 28 weeks gestational age) infants are significantly less likely than other VLBW and VP infants to receive mother’s own milk (MOM) through to discharge from the neonatal intensive care unit (NICU). The costs associated with adhering to pumping maternal breast milk are borne by mothers and contribute to this disparity. This randomized controlled trial tests the effectiveness and cost-effectiveness of an intervention to offset maternal costs associated with pumping.

**Methods:**

This randomized control trial will enroll 284 mothers and their VP infants to test an intervention (*NICU acquires MOM*) developed to facilitate maternal adherence to breast pump use by offsetting maternal costs that serve as barriers to sustaining MOM feedings and the receipt of MOM at NICU discharge. Compared to current standard of care (*mother provides MOM*), the intervention bundle includes three components: a) free hospital-grade electric breast pump, b) pickup of MOM, and c) payment for opportunity costs. The primary outcome is infant receipt of MOM at the time of NICU discharge, and secondary outcomes include infant receipt of any MOM during the NICU hospitalization, duration of MOM feedings (days), and cumulative dose of MOM feedings (total mL/kg of MOM) received by the infant during the NICU hospitalization; maternal duration of MOM pumping (days) and volume of MOM pumped (mLs); and total cost of NICU care. Additionally, we will compare the cost of the NICU acquiring MOM versus NICU acquiring donor human milk if MOM is not available and the cost-effectiveness of the intervention (*NICU acquires MOM*) versus standard of care (*mother provides MOM*).

**Discussion:**

This trial will determine the effectiveness of an economic intervention that transfers the costs of feeding VLBWand VP infants from mothers to the NICU to address the disparity in the receipt of MOM feedings at NICU discharge by Black infants. The cost-effectiveness analysis will provide data that inform the adoption and scalability of this intervention.

**Trial registration:**

ClinicalTrials.gov: NCT04540575, registered September 7, 2020.

## Background

In the United States, the burden of very low birth weight (VLBW; < 1500 g birth weight) birth is borne disproportionately by Black (non-Hispanic Black/African American) mothers, who are 2.2–2.6 times more likely than non-Black (non-Hispanic White, Hispanic, Asian) mothers to deliver VLBW infants [1]. This disparity affects Black families not only during the average 73-day hospitalization in the neonatal intensive care unit (NICU) [2] but continues after NICU discharge [3–10]. VLBW infants are susceptible to potentially preventable morbidities during the NICU hospitalization that increase the risk of costly lifelong health and neurodevelopmental problems [11–14]. Mother’s own milk (MOM; excludes donor human milk (DHM)) feedings received in the NICU reduce the risks and associated costs of several neonatal morbidities and neurodevelopmental problems in VLBW infants [9, 15–20]. However, Black VLBW infants are significantly less likely to receive MOM feedings from birth until NICU discharge than non-Black infants, which precludes exclusive MOM feedings for the first 6 months of life recommended by authorities [21, 22], further amplifying disparity of prematurity in the Black population.

Although Black mothers of VLBW infants initiate lactation at rates comparable to non-Black mothers and have similar goals to sustain MOM provision through to NICU discharge [23, 24], Black VLBW infants are significantly less likely to receive MOM at NICU discharge [2, 24–39]. Maternal adherence to a pumping regimen is required to sustain MOM provision, for which there are out-of-pocket and opportunity costs that are especially onerous for Black mothers, who are more likely to live in poverty [40]. In previous work, we found the difference between MOM receipt rates at NICU discharge for Black (23%) versus non-Black (43%) VLBW infants is mediated by poverty in Black mothers, despite these same mothers having comparable rates of MOM initiation and 87% indicating they wanted to provide MOM at NICU discharge [2, 23, 24, 39]. In contrast, when MOM is not available, the NICU provides pasteurized DHM and/or preterm formula, a current standard of care that subsidizes nutritionally inferior and significantly less protective milk feedings for this vulnerable population [41].

### Barriers to breast pump use by mothers of VLBW infants: pump

In the United States, breast pumps provided by public nutrition and health programs typically do not meet minimum criteria of effectiveness, efficiency and comfort required for breast pump dependency and are intended instead to supplement MOM removal for breastfeeding mothers of term infants during brief separations such as return to employment [42, 43]. High-quality, hospital-grade electric breast pumps that permit simultaneous breast emptying are available, but the mother is often required to pay out-of-pocket for the rental costs. Ineffective, inefficient and uncomfortable breast pumps contribute to lack of adherence to consistent, frequent breast pump use, especially during the first 14 days postpartum, a critical period that includes achievement of secretory activation and coming to volume (≥500 mLs of MOM per day by postpartum day 14), both of which strongly predict receipt of MOM at NICU discharge [42, 44–47]. This time period coincides with the mother’s transition from hospital to home, often unwell herself and leaving behind a VLBW infant in the NICU. Added to this burden is that low-income mothers of VLBW infants may encounter a several-day delay before they can access an inferior pump for in-home use because they must either travel to a government social support agency office or wait for mail delivery [42, 44, 48, 49].

### Barriers to breast pump use by mothers of VLBW infants: opportunity cost of providing breast milk

In addition to having access to an appropriate hospital-grade electric breast pump, mothers must adhere to a regimen of sustained breast pump use (6–8 times/day) for the entire NICU hospitalization. This regimen takes approximately 120 min daily [44]. This time represents opportunity costs borne by mothers, including forgoing paid and unpaid work to pump and store MOM and sanitize breast pump equipment.

### Barriers to transporting pumped breast milk to the NICU

Additional out-of-pocket and opportunity costs are incurred to transport MOM pumped in the home to the NICU, and Black mothers are less likely to have access to a car than non-Black mothers [2, 50]. Data also indicate that Black mothers and family members have significantly fewer visits to the NICU compared to non-Black mothers and family members, and the frequency of visits is positively associated with infant receipt of MOM at NICU discharge [51]. For low-income women, these costs represent a greater proportion of overall income, and they must balance the perceived value of adherence against priorities that may be more immediate such as food and childcare expenses.

To address the maternal costs that serve as barriers to sustaining MOM feedings, we designed the Reducing Disparity in Receipt of Mother’s Own Milk in Very Low Birth Weight Infants (ReDiMOM) single-center randomized controlled trial, which tests an economic bundle (*NICU acquires MOM*) developed to offset these maternal costs. The *NICU acquires MOM* 3-part intervention bundle includes: 1) free hospital-grade electric pump for in-home use; 2) pickup of MOM by a hospital employee trained in safe handling and transport of MOM; and 3) payment for opportunity costs of time spent pumping. This intervention is compared to current standard of care, *Mother provides MOM*, in which the mother assumes these costs.

This innovative economic intervention bundle is based on principles of conditional cash transfers (CCTs). While CCTs have and are currently being investigated in term populations to increase lactation rates [52, 53], they have not been tested as an intervention to increase MOM provision by mothers of hospitalized very preterm (VP, < 32 weeks gestational age (GA), inclusive of extremely preterm (EP), < 28 weeks GA) infants. Data from an RCT in term infants demonstrate that the use of a CCT resulted in higher rates of any but not of exclusive breastfeeding at 6–8 weeks post-birth but had no effect on mothers’ decisions to initiate lactation [52]. Qualitative findings suggest that mothers perceive CCTs to have value for breastfeeding, compensating for ongoing breastfeeding challenges and facilitating achievement of targeted breastfeeding milestones [54]. Related research on the use of CCTs for breastfeeding in healthy but primarily disadvantaged populations reveals that 1) mothers value cash or cash equivalents more than other financial incentives such as grocery vouchers or baby supplies [54, 55] and 2) healthcare providers are generally positive but cautious about choosing the proper incentive and monitoring that its use does not impact other family or provider relationships [56–60].

The objectives of the ReDiMOM trial are to 1) compare infant and maternal MOM outcomes for the *NICU acquires MOM* intervention group versus *mother provides MOM* control group; 2) compare the cost of the NICU acquiring MOM versus the NICU acquiring DHM as supplemental feedings when MOM volume is insufficient; and 3) compare the cost-effectiveness of *NICU acquires MOM* versus *mother provides MOM* for maternal and infant outcomes.

## Methods/design

### Setting

This stratified, randomized controlled trial takes place in the NICU at Rush University Medical Center (RUMC), a level III Chicago perinatal center with over 2000 primarily high-risk births and over 100 VP, VLBW infants admitted to the NICU annually. The RUMC NICU has 72 private patient rooms and serves a racially and ethnically diverse group of families. Each infant’s room is equipped with a hospital-grade breast pump for use by the mother when visiting the NICU and a locked refrigerator for MOM storage, with excess MOM stored in large industrial freezers dedicated to MOM in the NICU. Lactation care is provided in the NICU through a unique model with nearly all direct lactation care provided by breastfeeding peer counselors (BPCs), who are mostly mothers of former RUMC NICU infants [61].

### Eligibility criteria

At study initiation (December 2020), maternal inclusion criteria were: 1) delivery of a VLBW infant at RUMC, 2) age ≥ 18 years, 3) willing and able to share a valid Social Security number, 4) fluent in English or Spanish, and 5) having plans to pump to provide MOM to her infant. Infant inclusion criteria were: 1) birth GA < 32 0/7 weeks, 2) birth weight < 1500 g, 3) no significant congenital anomalies or chromosomal defects, and 4) < 96 h of age at enrollment. After enrollment of the first 20 mothers, inclusion criteria were modified (August 2021) to remove the infant birth weight limitation. This was due to lower than anticipated enrollment and exclusion of VP infants with birth weight > 1500 g, which is well within the normal weight for an infant born at this gestation [62], and because lactation challenges in mothers of VP infants are primarily dependent on GA which corresponds to incomplete maturation or secretory differentiation of the mammary gland based on the duration of pregnancy. The eligibility criteria applied to mothers and infants, and modification was approved by the RUMC Institutional Review Board and the National Institutes of Health.

Exclusion criteria include: maternal health conditions that are incompatible with MOM provision per the clinical judgment of the NICU attending physician and one of the multi-PIs (ALP), mother has participated in ReDiMOM with a previous pregnancy, mother is enrolled in another study that impacts lactation, in the neonatologist’s opinion the infant is unlikely to survive, or mother is COVID-19 positive at time of delivery.

### Informed consent

Mothers and their VP infants are included. Although pregnant women likely to deliver an infant with a GA < 32 0/7 weeks may be approached prenatally in order to discuss the study, written consent from the infant’s mother is obtained by study staff (grant project manager or research nurse) after the infant is born and eligibility criteria are confirmed. Enrollment must be completed by 96 h of age of the infant, a time window that accommodates mothers who may have perinatal complications and cannot be approached ethically prior to this time. The study protocol (18060410-IRB01) was initially approved by the RUMC Institutional Review Board (FWA00000482) on January 17, 2019.

### Randomization procedure

Immediately after enrollment, mothers complete a brief questionnaire to collect GA and maternal race and ethnicity. Then, mothers are randomized to one of two groups (control – *Mother provides MOM* vs. intervention – *NICU acquires MOM*) in a 1:1 fashion using a stratified random assignment procedure contained in Research Electronic Data Capture (REDCap) [63] and a randomization table created by the study biostatistician. Six strata have been created by crossing 1) two levels based on GA (extremely preterm < 28 weeks vs. very preterm 28–31 weeks) with 2) three racial and ethnic categories (Non-Hispanic Black, Hispanic, and Non-Hispanic White/other). Within strata, condition is randomized within randomly ordered blocks of four and six participants, an approach that optimizes balance in sample sizes across conditions while also minimizing ability to deductively determine the condition of any upcoming participant. Randomization is performed by the study staff member using REDCap in the presence of the mother, ensuring that both mother and study staff member witness the assignment simultaneously. Because the unit of randomization is the mother, infants who are members of a set of multiples are allocated to the same group.

### Control and intervention groups

All enrolled mothers do the following: 1) complete a W-9 form, including Social Security number for opportunity cost payment and study incentives; 2) maintain an electronic or paper pumping log for as long as they pump during the infant(s)’ NICU stay; 3) allow all of their pumped MOM to be weighed in the NICU; 4) complete study questionnaires via REDCap or with a member of the research team (Table 1); 5) provide a baseline MOM sample (2 mL) while pumping in the presence of a research team member or NICU nurse in the first few days post-delivery, which serves as a reference for later MOM samples to assure later MOM is from the same mother (see Milk Samples and Potential for Milk Adulteration); and 6) provide monthly milk samples of 5 mL while the infant is hospitalized.

**Table 1:** Study Questionnaires

Questionnaire	Frequency
Demographic	1x after randomization
Health and Lactation History	1x after randomization
Weekly (Health and Economic)	Weekly
COVID Impact	2x, 21 days and prior to discharge
Pumping Log	Daily

All mothers receive standard lactation care including access to a free hospital-grade, double electric breast pump while hospitalized postpartum and access to a free hospital-grade, double electric breast pump for use at their infant(s)’ bedside in the NICU, as detailed in Table 2. In addition to standard lactation care, mothers assigned to the control group have the option to self-pay for the rental of a hospital-grade breast pump for home use at a subsidized rate. Mothers assigned to the intervention group receive the same standard lactation care as mothers in the control group with the following additions: 1) a hospital-grade electric smart breast pump (Medela Symphony PLUS® Breast Pump, Medela LLC, McHenry, IL) for home use at no charge to the mother while the infant is in the NICU and the mother continues to pump; 2) free pickup of pumped MOM from the mother’s home 2–3 times/week as needed; and 3) payment for pumping and handling her milk at the January 2020 Illinois minimum wage of $9.25/h for 120 min per day for each day that the mother pumps during her infant’s NICU hospitalization.

**Table 2:** Study Components for Intervention and Control Groups

Component	Control mothers	Intervention mothers
Initiation of pumping in labor and delivery within 3 h post-delivery	X	X
Instruction in MOM expression, pump cleaning and milk storage by NICU breastfeeding peer counselors (BPCs)	X	X
Hospital-grade breast pump for use during postpartum hospital stay	X	X
Hospital-grade breast pump stored in the infant’s room for use during NICU visits	X	X
Free sterile MOM containers and bar code labels for in-NICU and home use	X	X
Storage of MOM in infant’s NICU room refrigerator and dedicated freezers in the NICU	X	X
Proactive daily monitoring by a BPC for the critical first 14 days postpartum followed by weekly contact with a BPC to discuss lactation needs and feeding goals	X	X
Access to BPCs from 7:00 am - 8:30 pm, 7 days a week	X	X
Support for direct breastfeeding	X	X
Rental of a hospital grade breast pump for home use – at mother’s cost at a subsidized rate	X	
Hospital-grade electric smart breast pump for home use while the infant is in the NICU and the mother continues to pump – at no charge to the mother		X
Pickup of expressed MOM from mother’s home 2–3 times/week during weekdays as needed – at no charge to the mother		X
Payment for pumping at the January 2020 Illinois minimum wage of $9.25/h for 2 h/day for each day that the mother pumps during her baby’s NICU stay		X

### NICU acquires MOM intervention logistics

All study mothers receive a tablet to complete study questionnaires during the study, after which they are given the tablet for personal use. SMART breast pumps with data loggers that self-measure and store pumping start and stop times are used for all subjects in the intervention group, both in-hospital and at home. These stored data enable accurate and objective calculation of the number of pumping sessions and total minutes spent pumping. SMART pump data are automatically uploaded from the pump to the tablet, then transmitted via cellular data service to the secure study database using Bluetooth technology. In the case that the Bluetooth data transfer process malfunctions for an individual pumping(s) session, a back-up manual transfer protocol using a USB flash drive in the SMART pump to access stored data is used.

MOM pickup is performed by a milk courier, a security-cleared research team member who has been trained in safe handling and transport of MOM. As needed, the milk courier collects individual mothers’ MOM up to 3 times weekly and delivers it to the RUMC NICU. During the COVID-19 pandemic, MOM pickup is contactless. In brief, mothers place the MOM bottles in the same state (fresh/refrigerated or frozen) in study-provided plastic bags. An electric portable freezer and a larger cooler with ice packs are in the pick-up vehicle to maintain milk in refrigerated or frozen status. MOM temperature is monitored using the digital display on the electric freezer and a thermocouple thermometer and probe in the cooler (Fluke; Everett, WA). The milk courier records the temperature in a paper log at the beginning of the shift and at every stop. Additionally, the number of bottles and MOM state (fresh/refrigerated or frozen) are recorded in the log at pick-up and delivery to the NICU.

Payment is made on a weekly basis using rechargeable debit cards based on the number of days pumping was recorded by the in-hospital or home SMART pumps. The rechargeable debit cards are HIPAA compliant and developed specifically for clinical trials and research studies, requiring no protected health information to be shared with the vendor (CT Payer, Tampa, Florida).

### Outcomes

The primary outcome is the infant’s receipt of MOM at NICU discharge, determined from the medical record for the last full day of hospitalization and categorized as “Yes” if the infant received exclusive or any MOM, and “No” if the infant received only formula. Receipt of MOM may be via direct breastfeeding or bottle, per the mother’s preference and availability. RUMC NICU standard lactation care includes education and support for direct breastfeeding for all mothers of hospitalized infants. VP infants in the RUMC NICU transition from DHM to formula at 33–34 weeks corrected GA, so infants only receive MOM or formula at the time of NICU discharge.

Secondary outcomes include the following: infant receipt of any MOM (any or none) during the NICU hospitalization, duration of any MOM feedings (days with any MOM feeding), and cumulative dose (total mL/kg) of MOM feedings received by the infant during the NICU hospitalization; maternal duration of MOM pumping (days) and volume of MOM pumped (mLs); and total cost of care, all determined at the time of infant discharge from the NICU. Duration of MOM pumping is calculated as the number of days the mother pumped any MOM. Total cost of care includes program, healthcare system and participant (maternal and infant) costs. Program costs include the cost of the intervention (rental costs for pumps used at home, milk courier time and mileage, supplies required for MOM pick up, opportunity cost payment to mothers). Healthcare system costs include costs borne by healthcare providers or third-party payers, such as the cost of the hospital stay, DHM and formula (Table 3). Participant costs include the opportunity cost of pumping and delivering MOM to the NICU (in the control group) and other participant and caregiver informal health sector and non-health sector out-of-pocket costs, such as travel costs to and from the NICU and the cost of care for other children during visits to the NICU.

**Table 3:** Cost Components

Resources	Units	Source	Cost Measure
**PROGRAM COSTS**			
STANDARD CARE			
Freezer for pumped MOM storage	Number	Finance	Purchase price
Human milk waterless warmer	Number	Finance	Purchase price
Creamatocrit	Number	Finance	Purchase price
Infant scale	Number	Finance	Purchase price
Liners for waterless warmer	Number per mother	Finance	Purchase price
Education materials	1 set per mother	Finance	Purchase price
Pump kit	1 per mother	Finance	Purchase price
Custom-fitted breast shields	1 per mother	Finance	Purchase price
Hospital grade HM storage containers	Number per mother	Finance	Purchase price
Lactation support (Breastfeeding Peer Counselor [BPC])	Hours per mother	T&M	Average hourly wage
‘NICU ACQUIRES MOM’ GROUP			
Hospital-grade breast pump	Months used	Study	Monthly leasing cost
Time to provide breast pump to mother (NICU Nurse)	Hours per mother	T&M	Average hourly wage
Transportation of MOM	Number of trips	Study	Standard IRS mileage rate
Time to transport MOM (Milk Courier)	Hours	Study	Hourly wage x number of trips
Personal coolers	2 per mother	Study	Purchase price
Opportunity cost payment	Number of days pumped	Pump Log	$18.50 per day
Debit card for opportunity cost payment	Weeks	Study	Set-up cost + (loading cost x number of weeks mother receives any opportunity cost payment)
Time to track MOM received in NICU (BPC)	Hours	Study	Average hourly wage
Time to process weekly opportunity cost payment (Research Manager)	Hours	Study	Average hourly wage
Application development and maintenance for mobile pumping log (Analyst)	Hours	Study	Average hourly wage
‘MOTHER PROVIDES MOM’ GROUP			
Time to lease breast pump to mother (NICU Nurse)	Hours	Study	Average hourly wage
Time for monthly collection of breast pump leasing fee (NICU Nurse)	Hours	Study	Average hourly wage
Time to retrieve leased breast pump at NICU discharge (NICU Nurse)	Hours	Study	Average hourly wage
**HEALTHCARE SYSTEM**			
NICU hospital stay	Resources used	Finance	Cost to Rush for hospital and providers to deliver care
DONOR HUMAN MILK			
mL of donor milk consumed	mL	EMR, Finance	Hospital purchase price per mL
Time to process and route DHM order (NICU Nurse)	Hours	T&M	Hourly wage
Shipping fees to NICU	Shipments and quantity per shipment	Finance	Hospital purchase price
Freezer DHM storage	Number	Finance	Purchase price annualized over 15 years / maximum quantity held / 365
Time to determine DHM amount to prepare, based on available MOM supply (Dietitian)	Hours	T&M	Hourly wage
NICU space to prepare DHM feedings to ensure safe handling	Square feet	Finance	NICU cost per square foot
Time to prepare DHM feeding (BPC)	Hours	T&M	Hourly wage
**PARTICIPANT COSTS**			
Time spent pumping	Time spent	Pump Log	Average hourly wage
Breast pump lease	Months	Study	Lease cost per month
Travel time to and from NICU	Time per trip; number of trips	Self-Report	Average hourly wage
Time spent in NICU	Number of visits; hours per visit	Self-Report	Average hourly wage
Transportation (e.g., public transportation, taxi, personal vehicle)	Number of trips	Self-Report	Cost per trip
Mother and other caregiver meals away from home	Number of meals	Self-Report	Cost per meal
Other out-of-pocket costs	Number	Self-Report	Cost per item

Notes: *MOM* mother’s own milk; *NICU* neonatal intensive care unit; *Finance* financial records; *T&M* time and motion study; *Study* study records; *Pump Log* maternal pumping log; *EMR* electronic medical record; *Self-Report* mother self-report

### Study visits and timeline

At enrollment, all mothers are given a study tablet with cellular service provided by the study and a CT Payer Visa debit card, loaded with an enrollment incentive of $20. Cellular service is discontinued at NICU discharge, and mothers keep the tablet at the end of the study. Intervention group mothers are also given a SMART pump at enrollment. All study questionnaires and data collection procedures can be completed remotely with the study tablet. Mothers randomized to the intervention group return the SMART pump when the infant is discharged from the NICU or when the mother discontinues pumping, whichever occurs sooner.

### Sample size

For the primary outcome, we estimated power based on expected rates of infants receiving MOM at NICU discharge of 33% in the control group vs 51% in the intervention group (OR = 2.1) using estimates from published U.S. studies that report MOM at discharge for VLBW infants [25, 34, 36, 64]. The published rates are generally higher than our assumptions, but these rates reflect much lower rates of socio-economic disadvantage than found in our population, because they represent statewide data [34, 64] and/or occur in higher socio-economic settings [25, 64]. Under these assumptions, using SAS Proc Power we calculated a required sample size of 256 mothers (128 per group) to have 80% power to detect an effect of OR = 2.1 or greater. To account for 10% attrition, we will enroll 284 mothers and their infants. All power analyses were conducted with α = .05, and 2-tailed tests.

### Data collection plan

Data are collected from several sources: 1) subject contact information form, 2) REDCap surveys, 3) data extraction from the electronic medical record and hospital financial system, 4) SMART breast pump and 5) measurement of pumped MOM volume. Data extracted from the electronic medical record include maternal and infant demographic characteristics and medical diagnoses, daily feeding volume (mL) and type (MOM, DHM, formula including name), daily receipt of mechanical ventilation, daily receipt of parenteral nutrition, and dates of hospitalization for infants. Coded numbers are used in association with data and samples to maintain confidentiality, and all records are maintained on a secure RUMC network drive that can only be accessed by study personnel using password protected login information.

Pumping duration is collected through mothers’ records of each pumping session duration in the REDCap Pumping Log or paper pumping logs that are transferred into REDCap by study staff. Volume of pumped MOM is collected by measuring exact weights (nearest 0.1 g) of empty and filled MOM storage containers using scientific scales (Tanita, Japan), then calculating MOM volume by subtracting the empty weight from the filled weight (1 g = 1 mL) [46, 65]. Filled containers are weighed by research staff, BPCs and/or bedside nurses when brought to the NICU, and weight and container size are recorded for later entry into study database.

Table 3 summarizes the program, healthcare system and participant (maternal and infant) resources and costs that will be collected. Detailed, micro-level cost data for the NICU hospital stay are retrieved from the RUMC financial system, which includes both hospital and physician resources used and their associated costs [15, 17]. Program resources and costs are collected via NICU financial records, study records, and direct observation of a subset of encounters to measure the duration of time for some activities (time and motion studies). All costs will be adjusted to 2020 US dollars using the Consumer Price Index for All Items, All Urban Consumers, from the US Bureau of Labor Statistics [66].

### Milk samples and potential for milk adulteration

An unlikely theoretical risk is the possibility that an intervention group mother would adulterate and/or replace her pumped MOM for infant feeding with a substitute, including milk from another lactating woman, to continue receiving payment for the opportunity costs of pumping. To address this concern, MOM from both groups is tested for adulteration at serial time points during the infant’s NICU hospitalization and mothers are informed about testing at the time of enrollment. The first 2 mL MOM sample is pumped in the presence of a research team member or NICU nurse during the mother’s maternity hospitalization or in the first few days post-delivery and serves as the reference sample, documenting maternal-specific DNA. Subsequent 2-5 mL MOM samples brought to the NICU are collected and tested randomly on a monthly basis. MOM samples remain frozen at -80C and will be shipped to The Ohio State University for testing by study co-investigator (JJK) in Year 5. Samples will be tested for the most common potential diluents for which analyses are available: infant formula (cow’s milk based), cow’s milk, goat’s milk, soy milk, almond milk, rice milk, and breast milk from another woman. 

### Statistical methods

All analyses will use an intent-to-treat approach in which participants will be analyzed according to the group to which they were assigned. All analyses will include terms for GA (< 28 weeks vs. 28–32 weeks), sex and racial and ethnic category (Black, Hispanic, White/other).

To test the effect of the intervention on infant receipt of any MOM at NICU discharge, a logistic regression model with treatment group and covariates previously described will be constructed. Similar regression models will be used to test secondary outcomes, with the model function (e.g., normal, ordinal, Poisson, time to event) appropriate to the distribution of the outcome being considered. In addition to the main effects of the intervention, secondary analyses will be conducted to test whether intervention effects vary as a function of infant GA and racial and ethnic category.

To compare the cost of the NICU acquiring MOM versus the NICU acquiring DHM as supplemental feedings when MOM volume is insufficient, the NICU cost per mL of acquiring MOM versus acquiring DHM will be calculated by multiplying the number of units of each resource (MOM or DHM) by its respective per-unit cost. The costs of the NICU acquiring MOM include all standard lactation care costs and the cost of the intervention components. The costs of the NICU acquiring DHM include the hospital’s purchase price of DHM and costs associated with processing and storing the DHM. The per-unit cost of acquiring MOM will be compared with the per-unit cost of acquiring DHM using a generalized linear regression model with a log link function and gamma distribution, including covariates previously described.

The cost-effectiveness analysis will compare *NICU acquires MOM* versus *mother provides MOM* and will be conducted from the societal perspective. The primary effectiveness measure will be receipt of MOM at NICU discharge, and additional analyses will be performed for the secondary outcomes. Cost-effectiveness will be calculated as the incremental cost-effectiveness ratio (ICER) for *NICU acquires MOM* relative to *Mother provides MOM*. Additional cost-effectiveness analyses will be conducted from the program, healthcare system and participant perspectives. The 95% confidence intervals using non-parametric estimation will be computed for the ICERs and cost-effectiveness acceptability curves derived from probabilistic sensitivity analysis will be plotted to summarize uncertainty. Cost-effectiveness analyses will follow best practices outlined by the Second Panel on Cost-Effectiveness in Health and Medicine [67].

### Data safety monitoring

An independent Data Safety Monitoring Board reviews adverse events and monitors data safety. Members of the Data Safety Monitoring Board include experts in the field of pediatrics, neonatology, biostatistics and clinical trials.

## Discussion

By employing an innovative economic-based strategy utilizing CCTs to overcome barriers faced by Black mothers, this randomized controlled trial tests whether adherence to pumping improves, as measured by whether a greater proportion of infants in the *NICU acquires MOM* intervention group receive MOM at the time of NICU discharge compared to infants in the *mother provides MOM* control group. We anticipate that the societal cost of the NICU acquiring MOM will be equal to or less than the cost of an equivalent volume of DHM feedings supplemented with formula due to the reductions in morbidities associated with MOM but not DHM and formula feedings. Scalability of the *NICU acquires MOM* intervention is informed by the fact that it is aligned with national and global recommendations and demand for strategies to increase MOM feedings in the VP infant population [68–70]. Despite the known benefits, disparities in MOM provision based on race, ethnicity and socioeconomic status are prevalent in US NICUs. Conceptualizing MOM feedings as an integral part of NICU care requires consideration of who bears the costs of MOM provision and testing novel interventions to offset costs traditionally borne by mothers, which are more onerous to low-income women [37, 71].

## Data Availability

Data will be available between 2 and 5 years after publication of the final study results to investigators whose proposed use of the data has been approved by an independent review committee identified for this purpose and deemed scientifically appropriate per the ReDiMOM principal investigators.

## Abbreviations

ReDiMOM: Reducing Disparity in Receipt of Mother’s Own Milk in Very Low Birth Weight Infants
VLBW: Very low birth weight (< 1500 g birth weight)
VP: Very preterm, < 32 weeks gestational age
MOM: Mother’s own milk
NICU: Neonatal intensive care unit
DHM: Donor human milk
CCTs: Conditional cash transfers
GA: Gestational age
EP: Extremely preterm, < 28 weeks gestational age
RUMC: Rush University Medical Center
BPC: Breastfeeding peer counselor
REDCap: Research Electronic Data Capture
